# Locus Coeruleus Degeneration Correlated with Levodopa Resistance in Parkinson’s Disease: A Retrospective Analysis

**DOI:** 10.3233/JPD-212720

**Published:** 2021-10-12

**Authors:** Cheng Zhou, Tao Guo, JingJing Wu, Linbo Wang, Xueqin Bai, Ting Gao, Xiaojun Guan, Luyan Gu, Peiyu Huang, Min Xuan, Quanquan Gu, Xiaojun Xu, Baorong Zhang, Wei Cheng, Jianfeng Feng, Minming Zhang

**Affiliations:** a Department of Radiology, The Second Affiliated Hospital, Zhejiang University School of Medicine, Hangzhou, China; b Institute of Science and Technology for Brain-Inspired Intelligence, Fudan University, Shanghai, China; c Department of Neurology, The Second Affiliated Hospital, Zhejiang University School of Medicine, Hangzhou, China; d Key Laboratory of Computational Neuroscience and Brain-Inspired Intelligence (Fudan University), Ministry of Education, Shanghai, China; e Department of Computer Science, University of Warwick, Coventry, United Kingdom

**Keywords:** Parkinson’s disease, locus coeruleus, levodopa, magnetic resonance imaging, network

## Abstract

**Background:**

The widely divergent responsiveness of Parkinson’s disease (PD) patients to levodopa is an important clinical issue because of its relationship with quality of life and disease prognosis. Preliminary animal experiments have suggested that degeneration of the locus coeruleus (LC) attenuates the efficacy of levodopa treatment.

**Objective:**

To explore the relationship between LC degeneration and levodopa responsiveness in PD patients *in vivo*.

**Methods:**

Neuromelanin-sensitive magnetic resonance imaging (NM-MRI), a good indicator of LC and substantia nigra (SN) degeneration, and levodopa challenge tests were conducted in 57 PD patients. Responsiveness to levodopa was evaluated by the rates of change of the Unified Parkinson’s Disease Rating Scale Part III score and somatomotor network synchronization calculated from resting-state functional MRI before and after levodopa administration. Next, we assessed the relationship between the contrast-to-noise ratio of LC (CNR_LC_) and levodopa responsiveness. Multiple linear regression analysis was conducted to rule out the potential influence of SN degeneration on levodopa responsiveness.

**Results:**

A significant positive correlation was found between CNR_LC_ and the motor improvement after levodopa administration (*R* = 0.421, *p* = 0.004). CNR_LC_ also correlated with improvement in somatomotor network synchronization (*R* = –0.323, *p* = 0.029). Furthermore, the relationship between CNR_LC_ and levodopa responsiveness was independent of SN degeneration.

**Conclusion:**

LC degeneration might be an essential factor for levodopa resistance. LC evaluation using NM-MRI might be an alternative tool for predicting levodopa responsiveness and for helping to stratify patients into clinical trials aimed at improving the efficacy of levodopa.

## INTRODUCTION

The widely diverse responsiveness of Parkinson’s disease (PD) patients to levodopa treatment is an important clinical issue because of its relationship with the quality of daily life and prognosis of the disease [1]. The mechanisms underlying this variability of responses to treatment are unclear. Despite the inter-subject variability in its pharmacokinetics, the degeneration of the locus coeruleus (LC)–nor-epinephrine system, which has significant interaction with the dopaminergic system, might potentially affect patient’s responsiveness to levodopa [2].

In addition to its association with several nonmotor symptoms, LC norepinephrine deficiency has a significant link with motor symptoms of PD [3]. Previous experiments in animals has suggested that loss of neurons in the LC enhances the neurodegeneration of the dopaminergic system and aggravates motor disturbance in PD patients [3, 4]. More importantly, preliminary evidence from a rat PD model showed that severe LC norepinephrine deficiency reduces the efficacy of levodopa treatment [2]. This result was also supported in mice treated with 1-methyl-4-phenyl-1,2,3,6-tetrahydropyridine (MPTP), whereby the motor-stimulating effect of levodopa was dramatically reduced in mice given additional LC lesions that reduce norepinephrine production [5]. The above-mentioned studies indicated that degeneration of the LC–noradrenergic system might be an important consideration when evaluating the effects of levodopa on PD patients. Further confirmation of the relationship between LC degeneration and the levodopa responsiveness of PD patients *in vivo* is of great importance for understanding the mechanism of levodopa resistance, and for stratifying PD patients into clinical trials.

A novel neuromelanin-sensitive magnetic resonance imaging (NM-MRI) technique, which is sensitive to the neuromelanin (paramagnetic in its iron-bound form) that accumulates in noradrenergic neurons, provides an opportunity to quantify the LC and substantia nigra (SN) *in vivo* [6–9]. The reduction of LC signal intensity in NM-MRI scans corresponds to the loss of noradrenergic neurons [6, 10, 11]. Therefore, in this study, we used the contrast-to-noise ratios of LC and SN (CNR_LC_ and CNR_SN_), which were obtained from NM-MRI scans to evaluate the degeneration of LC and SN *in vivo* [6, 12].

In addition to the improvement of motor performance, the normalization of impaired brain functional network organization might be another indicator of a patient’s responsiveness to levodopa [13]. Studies have shown that cortical functional connectivity is increased after levodopa administration, especially in the motor cortex [13–16]. It is known that the dysfunction of somatomotor cortex is an important underlying factor for the motor symptoms of PD [16], and that the somatomotor cortex is innervated by both dopaminergic and noradrenergic projections [17]. Therefore, we combined the improvement of both motor symptoms and somatomotor network synchronization as indicators of responsiveness to levodopa and assessed their relationships with the degeneration of the LC and SN.

We hypothesized that LC degeneration was independently correlated with levodopa responsiveness. Therefore, we assessed the relationship between MRI LC signal intensity with the improvement of 1) motor symptoms (part III of the Unified Parkinson’s Disease Rating Scale, UPDRS-III), and 2) somatomotor network synchronization after levodopa administration. Multiple linear regression analysis was conducted to rule out the influence of SN degeneration (an important factor in the pathophysiological process of PD).

## METHODS

### Participants

This study was approved by the Medical Ethics Committee of the Second Affiliated Hospital of Zhejiang University School of Medicine. All participants signed informed consent forms in accordance with the Declaration of Helsinki. We recruited 57 idiopathic PD patients from the Neurology Department, Second Affiliated Hospital of Zhejiang University School of Medicine and 65 healthy controls (HCs) between March 2019 and December 2020. PD was diagnosed by an experienced neurologist according to the UK Parkinson’s Disease Society Brain Bank diagnostic criteria. We excluded subjects with a history of head injury, neurological surgery, intracranial mass, cerebrovascular disorders, or other neurological and psychiatric diseases.

Disease severity was assessed using the UPDRS and Hoehn and Yahr scale. The UPDRS-III score and resting-state blood-oxygen-level-dependent (BOLD) MRI data were assessed during the OFF state (at least 12 h after withholding PD medications) and repeated 1 h after administration of 200 mg levodopa and 50 mg benserazide (ON state) [18]. Three-dimensional T1-weighted (3D T1) image data and NM-MRI data were obtained only during the OFF state. To further test our hypothesis, we divided the PD patients into two groups according to the ranking of their rate of change of UPDRS-III score (levodopa response group = 29, levodopa resistance group = 28). The difference in CNR_LC_ and CNR_SN_ between the two groups was assessed.

### Magnetic resonance imaging data acquisition

All imaging data were acquired from a 3-tesla MRI scanner (Discovery MR750, GE Healthcare; 8-channel head coil). The head of each participant was stabilized with foam pads, and earplugs were provided to reduce audible noise during scanning.

3D T1 images were acquired using a fast spoiled gradient-recalled sequence: echo time (TE) = 3.036 ms; repetition time (TR) = 7.336 ms; inversion time = 450 ms; flip angle (FA) = 11°; field of view (FOV) = 260×260 mm^2^; matrix = 256×256; slice thickness = 1.2 mm; number of slices = 196 (sagittal); scanning time = 5 min 53 s. The acquisition plane was parallel to the anterior commissure–posterior commissure (AC–PC) line.

Resting-state functional MRI (fMRI) data were acquired using a gradient-recalled echo–echo planar imaging sequence: TE = 30 ms; TR = 2000 ms; FA = 77°; FOV = 240×240 mm^2^; matrix = 64×64; slice thickness = 4 mm; slice gap = 0 mm; number of slices = 38 (axial); time points = 205; scanning time = 7 min. The acquisition plane was parallel to the AC–PC line.

NM-MRI was performed using a T1-weighted fast spin echo sequence: TE = 18.6 ms; TR = 600 ms; FA = 77°; FOV = 220×220 mm^2^; matrix = 512× 512; slice thickness = 3 mm; slice gap = 0 mm; number of slices = 17 (axial). The acquisition plane was orthogonal to the brainstem. Scanning coverage was set from the top of basal ganglia to the bottom of the medulla oblongata; scanning time = 10 min 27 s.

### CNR_*LC*_ and CNR_*SN*_ calculations

An author (C.Z.), who was blinded to the subjects’ information, performed two manual measurements with a time interval of one month. These measurements were processed using ITK-SNAP (https://sourceforge.net/projects/itk-snap/).

**Fig. 1:**
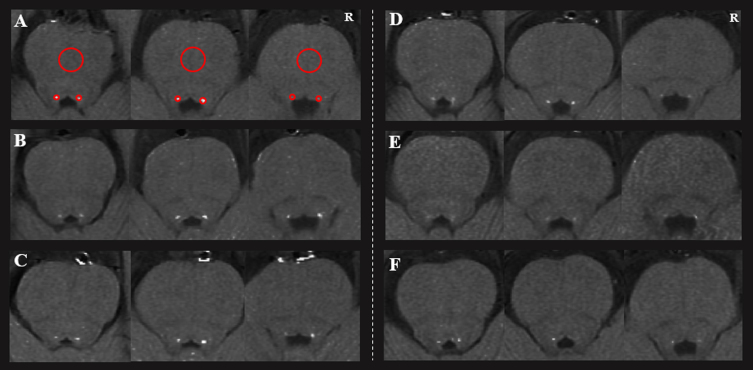
The loci coeruleus of three healthy controls (A, B, C) and three Parkinson’s disease patients (D, E, and F). Two regions of interest (red circles) were placed at the LC, with a larger one at the pons. R, right.

The LC was located in the bilateral areas of the dorsal pons symmetrically, adjacent to the fourth ventricle. Locations with highest signal intensity (SI) in three contiguous slices from the level of the inferior colliculi and extending to superior cerebellar peduncles were identified as LC. Circular regions of interest (ROIs) were demarcated in the bilateral LC and the midportions of the pons (PT) at the same slice (as a contrast area) [12]. The ROIs were placed at the predefined anatomical position of LC when the signal was significantly reduced. As shown in Fig. 1, the size of the ROIs was 2 mm^2^ for LC and 20 mm^2^ for PT [19]. The mean and standard deviation (SD) of the SI in bilateral LC and PT were calculated. The CNR_LC_ was calculated using the following equation: CNR_LC_ = (SI_LC_ –SI_PT_) / SD_PT_. Finally, the averaged CNR_LC_ value from three slices on right and left sides and two assessments were used for the final analysis. The intraclass correlation coefficient for the intra-rater agreement was 0.873.

Degeneration of the SN might be a necessary covariate because of its close relationship with the motor manifestations of PD [20]. Therefore, to remove the influence of SN degeneration, we calculated the CNR of the SN (CNR_SN_) by NM-MRI according to a previous method [21]. The SN was visible in three contiguous slices of NM-MRI scans. In the middle slice, which has the greatest area of SN, three ROIs with a size of 10 mm^2^ were placed at isometric lateral, central, and medial SN regions. The adjacent cerebral peduncle (CP) with a size of 30 mm^2^ was used as a contrast region (Supplementary Figure 1) [7]. The CNR_SN_ was calculated according to CNR_SN_ = (SI_SN_ –SI_CP_) / SD_CP_. The averaged CNR_SN_ was used for further analysis (six divisions: bilateral central, medial, and lateral SN parts in duplicate), because no significant difference was found between the bilateral SN (*p* = 0.541) [21]. The intraclass correlation coefficient for the intra-rater agreement was 0.875, which indicated a high consistency.

### fMRI data preprocessing

Resting-state fMRI images were preprocessed using the Data Processing and Analysis for Resting-State Brain Imaging tools (DPABI, http://rfmri.org/dpabi) based on Statistical Parametric Mapping 12 (https://www.fil.ion.ucl.ac.uk/spm). The first 10 time points were excluded from the analysis to allow for scanner stabilization and the participants’ adaptation to the environment. The remaining functional images were first corrected for within-scan differences in acquisition time between slices, followed by realignment to the middle volume to correct for interscan head motion. Subsequently, the processed images were registered to 3D T1 images and spatially normalized to a standard template (Montreal Neurological Institute). Corrected images were smoothed with a Gaussian kernel of 6×6×6 mm^3^ and then detrended. Nuisance covariates, including white matter, cerebrospinal fluid, and 24 motion parameters were regressed and temporal band-pass filtering was applied at a frequency range of 0.04–0.07 Hz, which is suitable for synchronization analysis [22]. Finally, subjects were excluded using the Jenkinson framewise displacement threshold of > 0.2 mm [23].

### Calculating the synchronization of the somatomotor network

In this study, we applied a phase-based synchronization analysis to characterize the organization of the functional network. The phase-based synchronization analysis is an appropriate method for evaluating the extent of network-level synchrony, and avoiding the curse of dimensionality and underestimation of the synchronicity [24, 25].

**Fig. 2:**
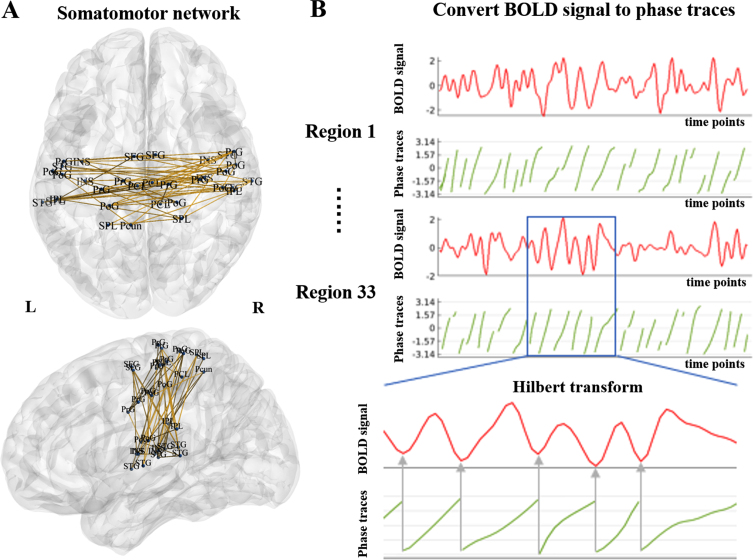
Diagram of the somatomotor network and the relationship between the BOLD signal and phase traces. A) Somatomotor network consisted of 33 nodes, including superior frontal gyrus (SFG), precentral gyrus (PrG), paracentral lobule (PCL), superior temporal gyrus (STG), superior parietal lobule (SPL), inferior parietal lobule (IPL), precuneus (Pcun), postcentral gyrus (PoG), and insular gyrus (INS). B) The BOLD signal was converted to phase traces using the Hilbert transform. BOLD: blood-oxygen-level dependent; L, left; R, right.


Processed fMRI data were used for phase-based synchronization analysis. We used the Brainnetome Atlas to construct functional networks of the brain [26]. This atlas was validated by both functional and structural anatomy and connectivity, consistent with the design and aims of the current study. For each subject, the regional time series of the somatomotor network [27] were extracted and represented as *s*_*j*_ (*t*), where *j* is the number of the somatomotor network’s nodes (*j* = 1, 2,  . . . , 33), and *t* is the number of time points (*t* = 1, 2,  . . . , 195). The Hilbert transform H[{*s*_*j*_ (*t*)] was applied to *s*_*j*_ (*t*) to obtain the associated analytical signals with instantaneous phase traces θ_*j*_ (*t*). The diagram of the somatomotor network and the relationship between the BOLD signal and phase traces are shown in Fig. 2.

(1) $$\theta_{j}\left(t\right)\;=\;\arctan\frac{H\left[s_{j}\left(t\right)\right]}{s_{j}\left(t\right)}$$

The first and last 10 time points were removed to minimize border effects inherent to the transform. Then, the mean phase synchrony of each time point r(*t*) was measured using the Kuramoto order parameter [28]:

(2) $$r\left(t\right)\;=\;\frac{1}{n}\left|\sum_{j=1}^{n}e^{i\theta_{j}\left(t\right)}\right|$$

where *n* = 33 is the total number of nodes.

Finally, we quantified the temporal averages of the somatomotor network synchronization with the mean order parameter <r(*t*)>:

(3) $$<r\left(t\right)>\;=\;\frac{1}{L}\sum_{t=1}^{L}r\left(t\right)$$

for which the total number of time points is *L* = 175. <r(*t*)>was used for the analysis that followed [22, 29–32].

### Statistical analysis

Statistical analysis was performed using the Statistical Package for the Social Sciences, version 22. A *p* value < 0.05 was considered as significant. Analysis of demographic and clinical data were assessed using two-sample *T*-test and chi-squared test, as appropriate. The relationships between CNR_LC_ and CNR_SN_ and the UPDRS-III score during the OFF and ON states were calculated. Age was regressed as a covariate of no interest.

The rate of change of UPDRS-III score was calculated as (UPDRS-III_OFF_ –UPDRS-III_ON_)/UPDRS-III_OFF_. The rate of change of somatomotor network synchronization was calculated as (Synchronization_OFF_ –Synchronization_ON_)/Synchronization_OFF_. Partial correlation analysis was conducted to assess the relationships among CNR_LC_, CNR_SN_, the rate of change of UPDRS-III score, the rate of change of somatomotor network synchronization. The relationships between CNR_LC_ and CNR_SN_ and the change of UPDRS-III score (original change of UPDRS-III score: UPDRS-III_OFF_ –UPDRS-III_ON_) were also assessed. Age, duration of dopaminergic drug administration, and levodopa equivalent daily dose (LEDD) were regressed as covariates of no interest. In addition, a stepwise multiple linear regression analysis was conducted to rule out the influence of SN degeneration. Potential factors including CNR_LC_, CNR_SN_, age, duration of dopaminergic drug administration, and LEDD were included.

## RESULTS

### Demographics, CNR_*LC*_, CNR_*SN*_, and levodopa responsiveness

No significant differences were found between HC subjects and PD patients in terms of age (*p* = 0.954), gender (*p* = 0.950), education (*p* = 0.758), or Mini-Mental State Examination (MMSE, *p* = 0.244). PD patients had significantly lower CNR_LC_ (*p* = 0.003) and CNR_SN_ (*p* < 0.001) values than HCs. Detailed demographic and clinical characteristics, CNR_LC_, and CNR_SN_ of HC and PD groups are shown in Table 1.

The two PD subgroups showed no significant differences in age (*p* = 0.497), gender (*p* = 0.912), education (*p* = 0.445), duration of drug administration (*p* = 0.695), LEDD (*p* = 0.728), UPDRS-I score (*p* = 0.810), UPDRS-II score (*p* = 0.563), UPDRS-III score (*p* = 0.118), Hoehn and Yahr stage (*p* = 0.734), or MMSE (*p* = 0.875). The levodopa response group showed significantly higher CNR_LC_ values than the levodopa resistance group (*p* = 0.007). No difference was found in CNR_SN_ between the two PD subgroups (*p* = 0.166).

**Table 1:** Demographic and clinical characteristics of HC and PD participants

	HC (65)	PD (57)	*p*
Age (y)	60.74±4.77	60.66±9.17	0.954
Gender (male/female)	38/27	33/24	0.950
Education (y)	8.46±3.28	8.18±4.46	0.758
Disease duration (y)	–	4.64±2.99	–
Duration of drug administration	–	3.78±3.34	–
LEDD (mg)	–	479.76±359.54	–
UPDRS-I	–	1.51±1.35	–
UPDRS-II	–	8.77±4.35	–
UPDRS-III (OFF)	–	22.12±12.99	–
UPDRS-III (ON)	–	14.21±10.75	–
Levodopa responsiveness	–	0.38±0.19	–
UPDRS-IV	–	1.07±1.65	–
Hoehn and Yahr stage	–	2.20±0.65	–
MMSE	27.80±1.92	27.23±3.35	0.244
CNR_LC_	1.66±0.66	1.34±0.49	0.003^*^
CNR_SN_	2.65±0.53	1.96±0.68	<0.001^*^

HC, healthy control; PD, Parkinson’s disease; LEDD, Levodopa equivalent daily dose; UPDRS, Unified Parkinson’s Disease Rating Scale; MMSE, Mini-Mental State Examination; CNR_LC_, Contrast-to-noise ratio of the locus coeruleus; CNR_SN_, Contrast-to-noise ratio of the substantia nigra.

### The relationships among CNR_*LC*_, CNR_*SN*_, and levodopa responsiveness

No direct correlation was found between the CNR_LC_ and UPDRS-III score during OFF (*R* = –0.040, *p* = 0.770) or ON states (*R* = –0.194, *p* = 0.152). On the contrary, the CNR_SN_ significantly correlated with the UPDRS-III score during OFF (*R* = –0.516, *p* < 0.001) and ON states (*R* = –0.482, *p* < 0.001). The corresponding correlation diagrams are shown in Supplementary Figure 2.

**Fig. 3:**
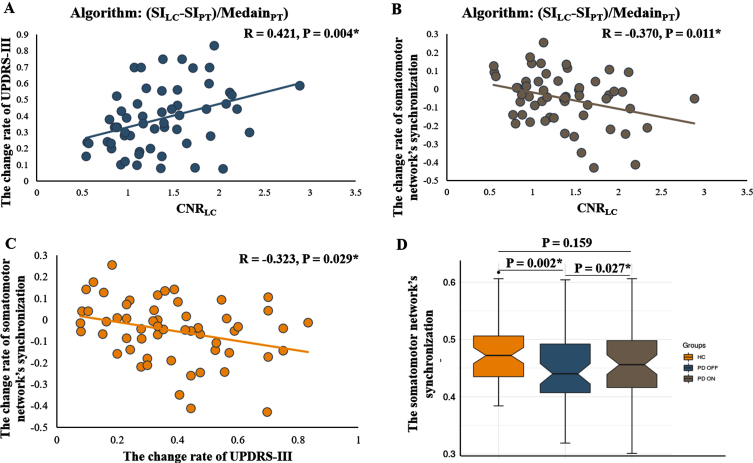
A-C) Partial correlation analysis between the CNR_LC_, the rate of change of UPDRS-III score, and the rate of change of somatomotor network synchronization in the PD group. Age, dopaminergic drug administration, and levodopa equivalent daily dose were regressed as covariates of no interest. The residuals of these are presented. D) The difference of somatomotor network synchronization among HC and PD subjects at OFF and ON states. CNR_LC_, contrast-to-noise ratio of LC.

The CNR_LC_ was positively associated with the rate of change of UPDRS-III score (*R* = 0.343, *p* = 0.009, and *R* = 0.421, *p* = 0.004 after regressing out covariates; Fig. 3A). The rate of change of UPDRS-III score was correlated with the synchronization of the somatomotor network (*R* = –0.317, *p* = 0.016, and *R* = –0.370, *p* = 0.011 after regressing out covariates; Fig. 3B). The CNR_LC_ was negatively associated with the rate of change of somatomotor network synchronization (*R* = –0.308, *p* = 0.020, and *R* = –0.323, *p* = 0.029 after regressing out covariates; Fig. 3C). No significant correlation was found between the rates of change of UPDRS-III score (*R* = –0.056, *p* = 0.710) or somatomotor network synchronization (*R* = 0.042, *p* = 0.780) and CNR_SN_ (Supplementary Figure 3).

PD patients showed reduced synchronization of the somatomotor network (Fig. 3D, *p* = 0.002) during the OFF state compared with HCs. No significant difference was found in the synchronization of the somatomotor network during the ON state when compared with HCs (Fig. 3D, *p* = 0.159). In PD patients, the synchronization of the somatomotor network was significantly increased after levodopa administration (Fig. 3D, *p* = 0.027).

In addition, a significant correlation was found between the CNR_LC_ and the change of UPDRS-III score (UPDRS-III_OFF_ –UPDRS-III_ON_; *R* = 0.346, *p* = 0.018). No correlation was found between the CNR_SN_ and the change of UPDRS-III score (*R* = –0.238, *p* = 0.111).

### Multiple linear regression analysis

Multiple linear regression analysis suggested that only CNR_LC_ (β= 0.423, *p* = 0.002), and not CNR_SN_ (β= –0.005, *p* = 0.972), age (β= –0.123, *p* = 0.360), duration of dopaminergic drug administration (β= –0.081, *p* = 0.545), or LEDD (β= 0.028, *p* = 0.833) could affect the rate of change of UPDRS-III score.

## DISCUSSION

In this study, we demonstrated a significant correlation between LC degeneration and responsiveness of PD patients to levodopa: the CNR_LC_ correlated with the rates of change of UPDRS-III score and somatomotor network synchronization. Multiple linear regression analysis showed that this relationship was independent of SN degeneration. To our knowledge, this is the first study assessing the relationship between LC degeneration and levodopa resistance in PD patients *in vivo*.

LC degeneration is one of the hallmarks of PD pathology. Whether a direct relationship exists between LC degeneration and motor disturbance is still a matter of controversy. Several studies have found a direct correlation between LC degeneration and cardinal motor symptoms of PD [3, 10, 33]. In the present study, although no direct correlation was found between LC degeneration and motor disturbance, LC signal intensity in MRI scans was significantly associated with improvement of UPDRS-III score. This relationship remained significant after removing the patients with a large daily dose of dopaminergic drugs (Supplementary Material). PD patients with weak responses to levodopa had more significant LC degeneration. The relationship between LC degeneration and levodopa resistance was also supported by results from a rat PD model, in which the motor recovery effects of levodopa were reduced in rats given an additional LC lesion [2]. Pharmacological studies have demonstrated that the existence of noradrenergic inputs facilitates dopaminergic transmission [34, 35], and the alpha-2 adrenoceptor antagonist atipamezole was shown to improve the efficacy of levodopa in an animal model of PD [36]. Recently, a clinical observational study reported that PD patients with severe rapid eye movement behavior disorder and autonomic dysfunction (symptoms closely associated with LC degeneration) [1, 10, 37] show less response to levodopa and rapid disease progression [1]. This phenomenon indirectly supported our findings. It should also be noted that perhaps LC degeneration is a hallmark of a more intractable PD subtype, but not a direct regulator of motor performance. Although such conclusions are outside the scope of the current study, future studies combining comprehensive clinical evaluation and bioinformatics might help to address this question. Considering the significance of the levodopa challenge test for predicting disease prognosis and the efficacy of deep brain stimulation treatment [38], this study indicated that CNR_LC_ might be a simple and promising alternative indicator, which was established on a pathological basis. In addition, the individual differences observed in the improvement of motor symptoms with noradrenergic agents highlight the need for stratification of PD patients in clinical trials [39, 40].

Research has suggested that the synchrony of cortical neuron activity has great significance for motor performance [41]. The relationship between the rates of change of UPDRS-III score and somatomotor network synchronization further support this viewpoint. The improvement of somatomotor network synchronization might be an indicator of levodopa responsiveness at the functional network level. It is known that the motor cortex is innervated by both dopaminergic and noradrenergic projections [17]. Experiments in rat models have demonstrated that noradrenergic terminals arising from the LC might be involved in modulating the functional activity of dopaminergic terminals in multiple cortical regions [42]. Meanwhile, LC norepinephrine might increase synaptic plasticity and facilitate new inputs [43, 44]. The above-mentioned evidence might explain the relationship between the response of somatomotor networks and LC degeneration. Our findings further validate the significance of LC norepinephrine in responsiveness to levodopa in the context of brain network organization.

Finally, considering the relationship between motor disturbance and SN degeneration, we also assessed the influence of SN degeneration on levodopa resistance. We found notable SN degeneration in PD patients, and the CNR_SN_ was significantly associated with the UPDRS-III score (in both OFF and ON states). This finding is highly consistent with those of previous studies, which indicates the good repeatability of the current study [21, 45]. Although SN degeneration was associated with severity of motor impairment, no direct association was found between SN degeneration and responsiveness to levodopa. This indicates that a non-dopaminergic factor is involved in levodopa response mechanisms. Multiple linear regression analysis further confirmed that the relationship between LC degeneration and levodopa resistance was independent of SN degeneration. Therefore, we considered that the severity of SN degeneration might not be a main driver of the responsiveness to levodopa.

The limitations of this study should be acknowledged. Because of the small size of LC, current image resolution might be prone to the partial-volume effect et al. Therefore, confirmation of the current findings using high- or ultrahigh-resolution NM-MRI would be desirable. The current sample size was relatively small owing to the difficulties and complexities of the levodopa challenge test and the multiple modalities of MRI data acquisition. A larger sample of patients is required in future studies to validate these findings. Finally, all PD patients were treated with levodopa, thus the magnitude of the acute test response might be affected by the long-term response to chronic levodopa therapy [46]. Future studies should aim to validate the present findings with NM-MRI in a drug-naïve PD cohort.

## CONCLUSIONS

We concluded that LC degeneration might be an essential factor for levodopa resistance. LC integrity evaluation using NM-MRI might be an alternative simple mean in evaluating the levodopa responsiveness and stratifying PD patients into clinical trials.

## Supplementary Material

Supplementary Material
