# Supplementary material for: Locus Coeruleus Degeneration Correlated with Levodopa Resistance in Parkinson’s Disease: A Retrospective Analysis
Source: J Parkinsons Dis. 2021 Oct 12;11(4):1631–40. doi: 10.3233/JPD-212720 (PMC8609680; doi:10.3233/JPD-212720)

Supplementary Materials

Locus Coeruleus Degeneration Correlated with Levodopa Resistance in Parkinson's Disease: A Retrospective Analysis

The coherence of different methods for CNR_{LC} calculation

We calculated the CNR_{LC} using another algorithm ($SI_{LC} – SI_{PT}$ / $Medain_{PT}$) to ruled out the influence of methodology. The CNR_{LC} calculated using two methods was highly consistent ($R = 0.881$, $p < 0.001$, Supplementary Figure 4A). This indicated that the two algorithms have good repeatability. And the relationships between CNR_{LC} ($SI_{LC} – SI_{PT}$ / $Medain_{PT}$) with the change rate of UPDRS-III ($R = 0.355$, $p = 0.015$, Supplementary Figure 4B) and somatomotor network synchronization ($R = -0.317$, $p = 0.032$, Supplementary Figure 4C) kept significant. This indicated that the two algorithms have good repeatability.

The coherence of different methods for CNR_{SN} calculation

In previous studies, different methods were used for calculating CNR_{SN}. To further validated our current findings, we calculated the CNR_{SN} using another method: round like ROIs for the SN were placed on 3 consecutive slices, starting from the most caudal slice with visible hyperintensity in the anatomic region of SN [1]. Three ROIs with a size of 10 mm^2 were placed at isometric lateral, central, and medial SN parts regions. The adjacent cerebral peduncles (CP) with a size of 30 mm^2 was used as a contrast region. The CNR_{SN} was calculated using the following equation: $CNR_{SN} = (SI_{SN} – SI_{CP}) / SD_{CP}$. Twice assessments were conducted by the

first author with a time interval of one week. Finally, the averaged CNR_{SN} was used for further analysis (twice assessments, three slices, six divisions: bilateral central, medial, and lateral SN parts).

The intraclass correlation coefficient value for the intra-rater agreement was 0.924. The two methods (one slice and three slices) showed a moderate to high consistency ($R = 0.709$, $p < 0.001$). PD patients showed significant reduced CNR_{SN} when compared with HCs (PD: 1.860±0.560, HC: 2.469±0.388, $p < 0.001$). The CNR_{SN} was significantly correlated with the UPDRS-III score during OFF ($R = -0.467$, $p < 0.001$) and ON states ($R = -0.421$, $p = 0.001$). No correlation was found between CNR_{SN} with the change rate of UPDRS-III score ($R = -0.128$, $p = 0.395$) and the change rate of somatomotor network synchronization ($R = -0.029$, $p = 0.847$). The relationships between CNR_{LC} with the change rate of UPDRS-III score ($R = 0.442$, $p = 0.002$), and CNR_{LC} with the change rate of somatomotor network synchronization ($R = -0.322$, $p = 0.031$) were significant after regressing the influence of CNR_{SN}. These results further confirmed our hypothesis that LC degeneration correlated with the levodopa resistance in PD patients.

Remove the potential influence of inadequate levodopa dose

To remove the potential influence of inadequate levodopa dose, we excluded the patients who have a signal dose ≥ 250 mg LEDD (n =11). Forty-six PD patient were enrolled for analysis. We assessed the relationships between the CNR_{LC} with the change rate of UPDRS-III and somatomotor network synchronization. Age, duration of dopaminergic drug administration, and LEDD were regressed as covariates of no interest. We found that the relationships between the

CNR_{LC} with the change rate of UPDRS-III ($R = 0.447$, $p = 0.003$) and somatomotor network synchronization ($R = -0.339$, $p = 0.040$) kept significant.

The relationship between the change rate of UPDRS-III with the left and right sides of CNR_{LC}

We calculated the CNR_{LC} of right and left sides separately. The CNR_{LC} of both left and right sides were significantly correlated with the change rate of UPDRS-III ($R = 0.398$, $p = 0.006$ for left side; $R = 0.377$, $p = 0.010$ for right side).

In addition, more than 80% patients (46/57) showed bilateral symptoms. No significant difference was found between right (8.40 ± 5.13) and left (7.00 ± 6.26) UPDRS-III score ($p = 0.186$). Therefore, the averaged CNR_{LC} was used in main text.

The relationship between the change rate of UPDRS-III with the left and right sides of CNR_{SN}

To avoid potential influence of the lateralization of SN degeneration on levodopa responsiveness, the relationships between left and right sides of CNR_{SN} and the change rate of UPDRS-III was assessed separately. No significant correlation was found between levodopa responsiveness with left CNR_{SN} ($R = -0.088$, $p = 0.563$) and right CNR_{SN} ($R = -0.040$, $p = 0.794$). This further confirmed that the relationship between CNR_{LC} and levodopa responsiveness was independent of SN degeneration.

The relationships between the synchronization of other brain networks, CNR_{LC}, and the change rate of UPDRS-III

In addition to the somatomotor network, the relationships between the change rate of other cortical network's synchronization with the change rate of UPDRS-III score (Supplementary Figure 6A), and CNR_{LC} (Supplementary Figure 6B) were further assessed. The difference of network synchronization among healthy controls, PD patients during OFF and ON were shown in Supplementary Figure 7.

REFERENCE

[1] Liu Y, Li J, He N, Chen Y, Jin Z, Yan F, Haacke EM (2020) Optimizing neuromelanin contrast in the substantia nigra and locus coeruleus using a magnetization transfer contrast prepared 3D gradient recalled echo sequence. *Neuroimage* **218**, 116935.

Supplementary Figure 1. CNR_{SN} calculation. Six small red circles were placed at the central, medial, and lateral part of bilateral SN, and twice large red circles were placed at the cerebral peduncles. R: L, Left side: Right side; HC, healthy control; PD, Parkinson’s disease.

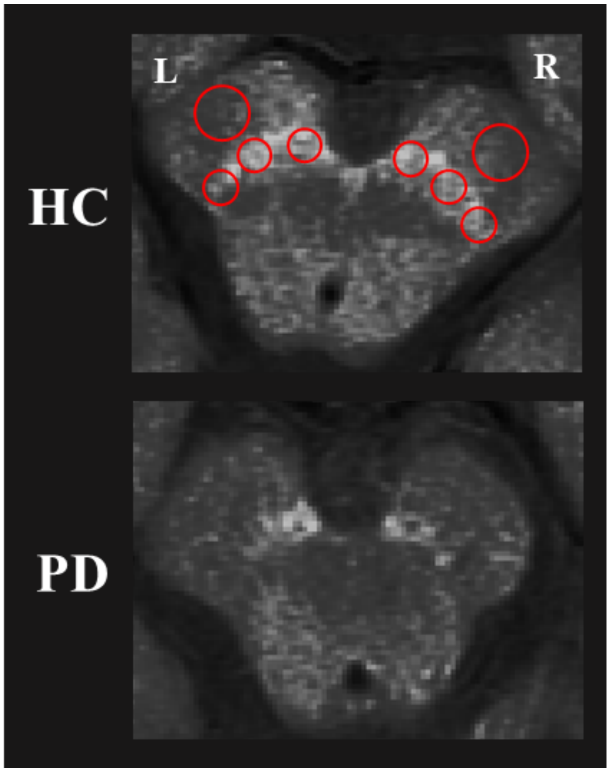

Supplementary Figure 2. A, B) The relationship between CNR_{LC} and the UPDRS-III score during OFF and ON state. C, D) The relationship between CNR_{SN} and the UPDRS-III score during OFF and ON state. CNR_{LC}, Contrast-to-noise ratio of the locus coeruleus; CNR_{SN}, Contrast-to-noise ratio of the substantia nigra; UPDRS-III, part three of Unified Parkinson's Disease Rating Scale.

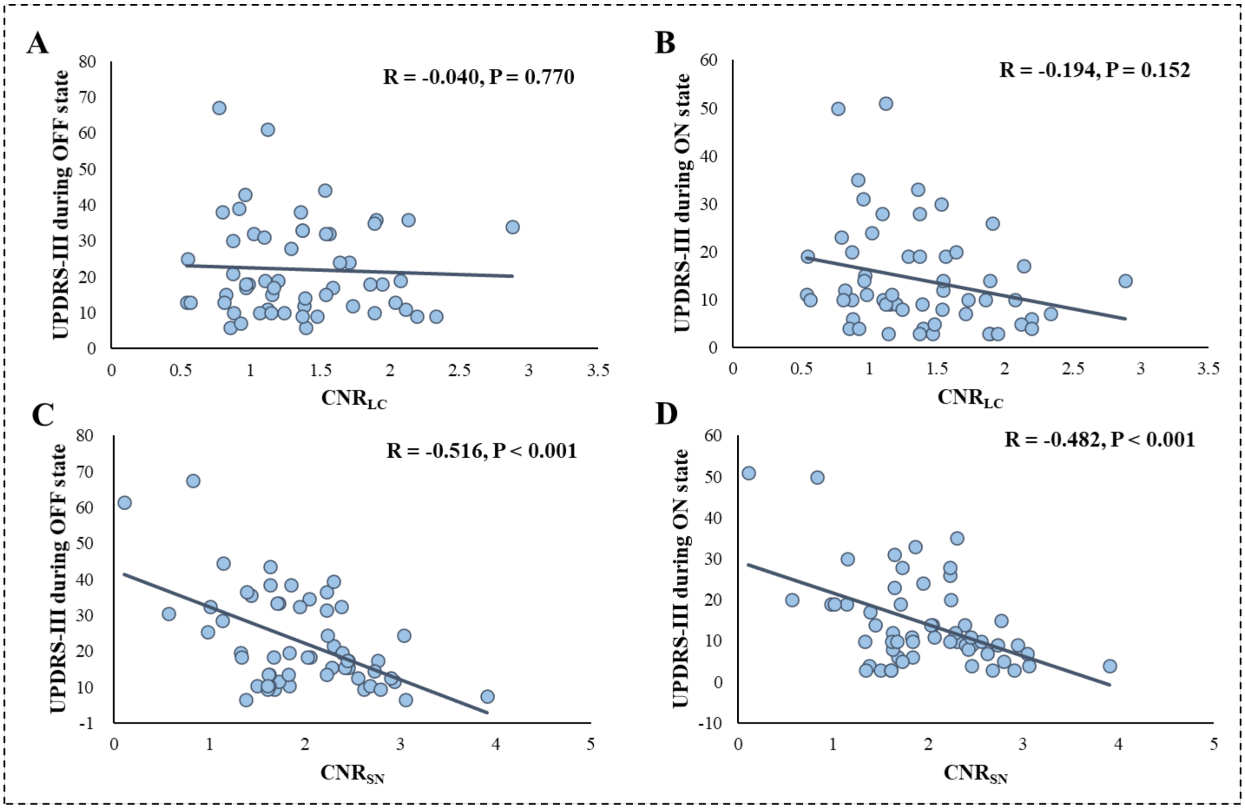

Supplementary Figure 3. The relationship between CNR_{SN} with the change rate of UPDRS-III and the change rate of somatomotor network synchronization. CNR_{SN}, Contrast-to-noise ratio of the substantia nigra; UPDRS-III, part three of Unified Parkinson's Disease Rating Scale.

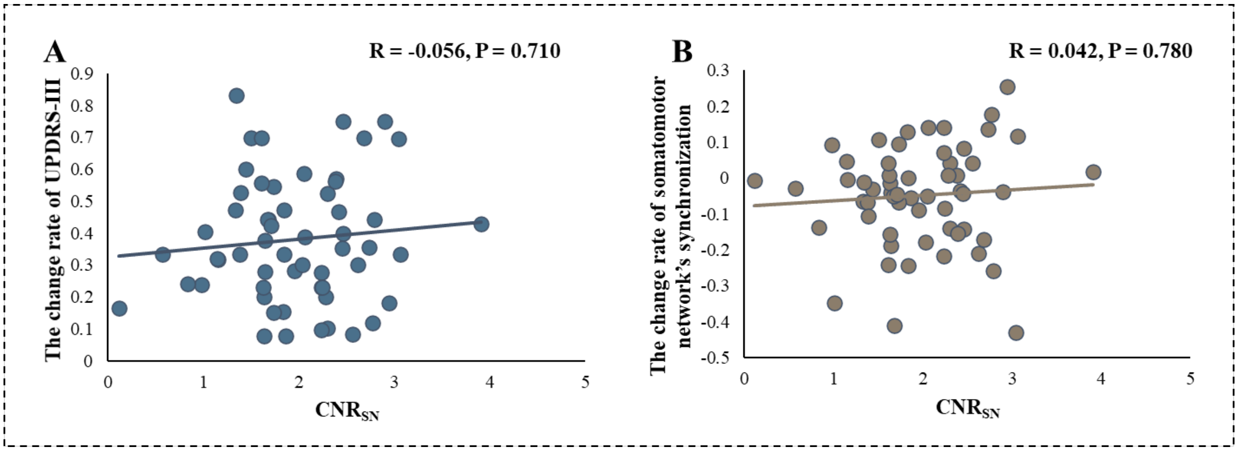

Supplementary Figure 4. A) The correlation between the CNR_{LC} calculated using two methods; B) The relationships between CNR_{LC} $(SI_{LC}-SI_{PT})/Medain_{PT}$) with the change rate of UPDRS-III; C) The relationships between CNR_{LC} $(SI_{LC}-SI_{PT})/Medain_{PT}$) with the change rate of somatomotor network synchronization. CNR, contrast-to-noise ratio; LC, locus coeruleus; SI, signal intensity; PT, pontine; SD, standard deviation; UPDRS-III, part three of Unified Parkinson's Disease Rating Scale.

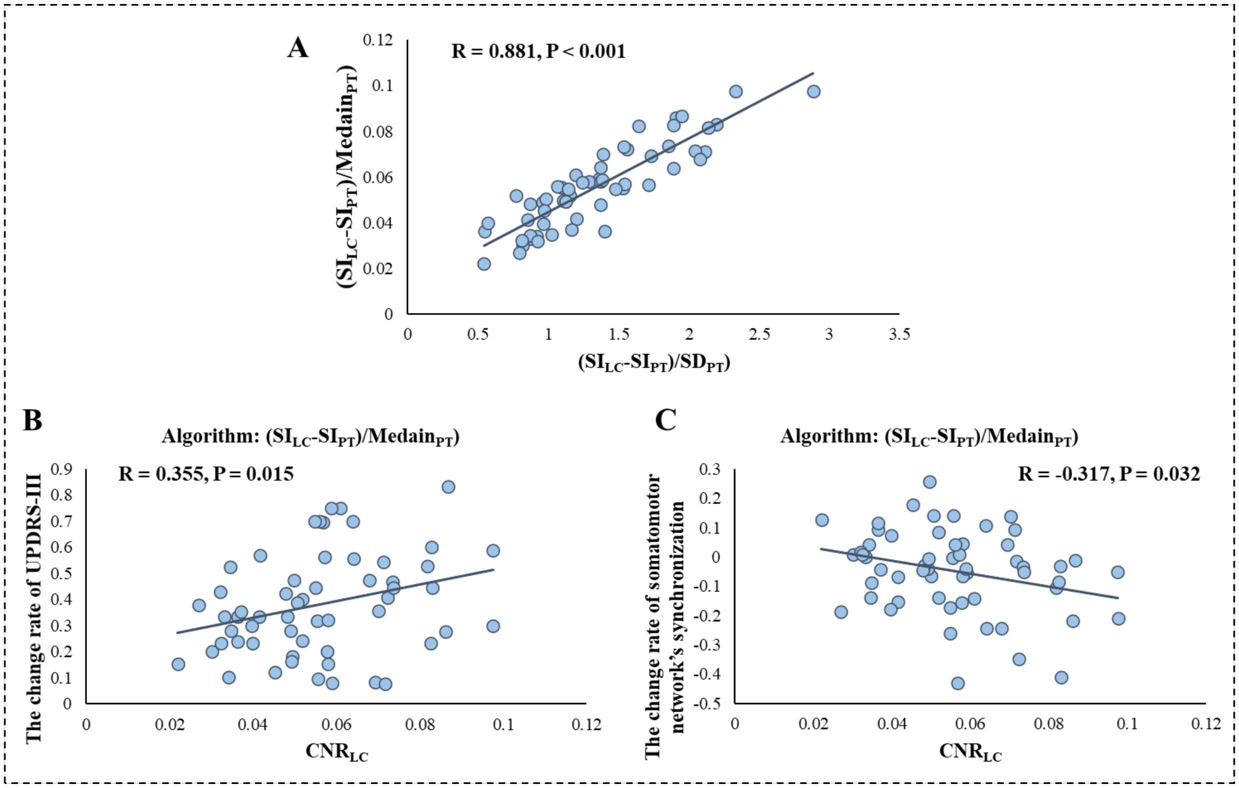

Supplementary Figure 5. A, B) The relationships between CNR_{SN} (three slices) and UPDRS-III score during OFF and ON; C) The relationships between CNR_{SN} and the change rate of UPDRS-III score; D) The relationships between CNR_{SN} and the change rate of somatomotor network synchronization. CNR_{SN}, Contrast-to-noise ratio of the substantia nigra; UPDRS-III, part three of Unified Parkinson's Disease Rating Scale.

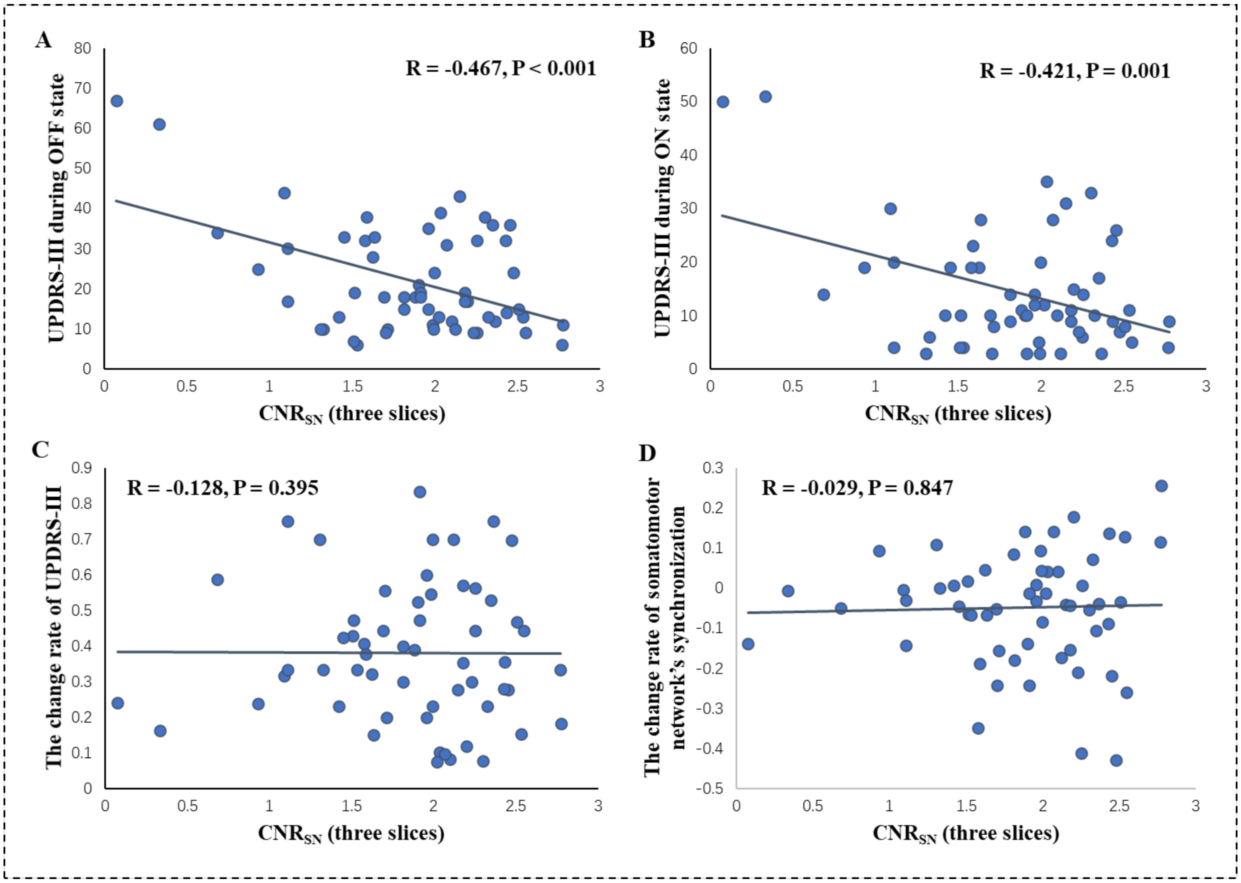

Supplementary Figure 6. The relationships between the synchronization of brain networks with the change rate of UPDRS III score and CNR_{LC}. A) In addition to the somatomotor network, the change rate of VN and DAN's synchronization were significantly associated with the change rate of UPDRS III scores. B) No significant correlation was found between CNR_{LC} and the improvement of non-motor network's synchronization. Uncorrected $p < 0.05$ was considered as significant. DAN, Dorsal attention network; DMN, Default mode network; FTP, Frontoparietal network; Lim, Limbic network; VAN, Ventral attention network; VN, Visual network. SNN, subcortical nucleus network.

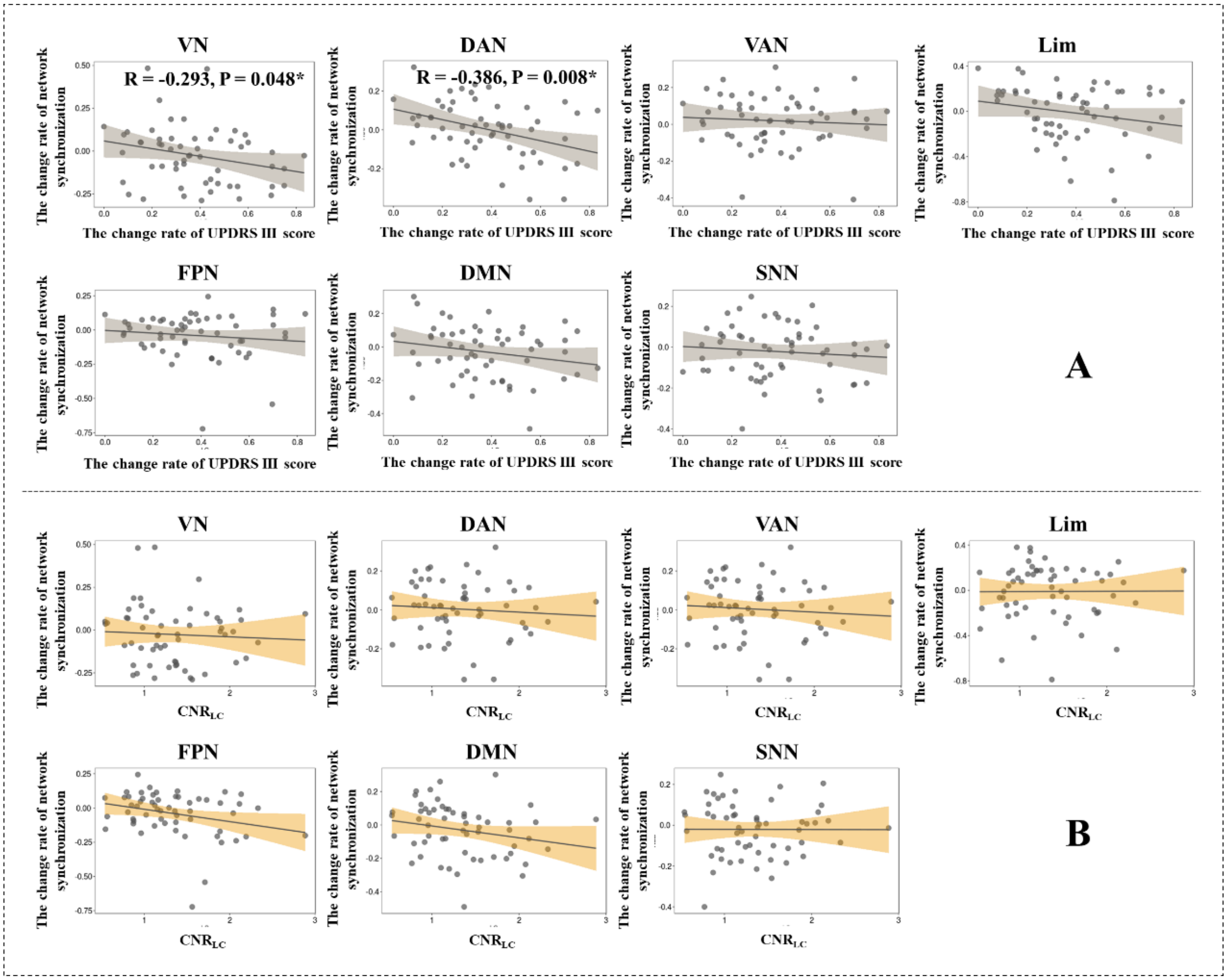

Supplementary Figure 7. The difference of network synchronization among healthy controls, PD patients during OFF and ON. In addition to somatomotor network, PD patients showed significant decreased synchronization in VN, DAN, VAN, FPN, DMN when compared with HC (healthy controls), which were consisted with previous studies. Except for somatomotor network, no significantly increased synchronization was found after levodopa administration. DAN, Dorsal attention network; DMN, Default mode network; FTP, Frontoparietal network; Lim, Limbic network; SN, Subcortical network; VAN, Ventral attention network; VN, Visual network; SNN, subcortical nucleus network. *p < 0.05; **p < 0.01.

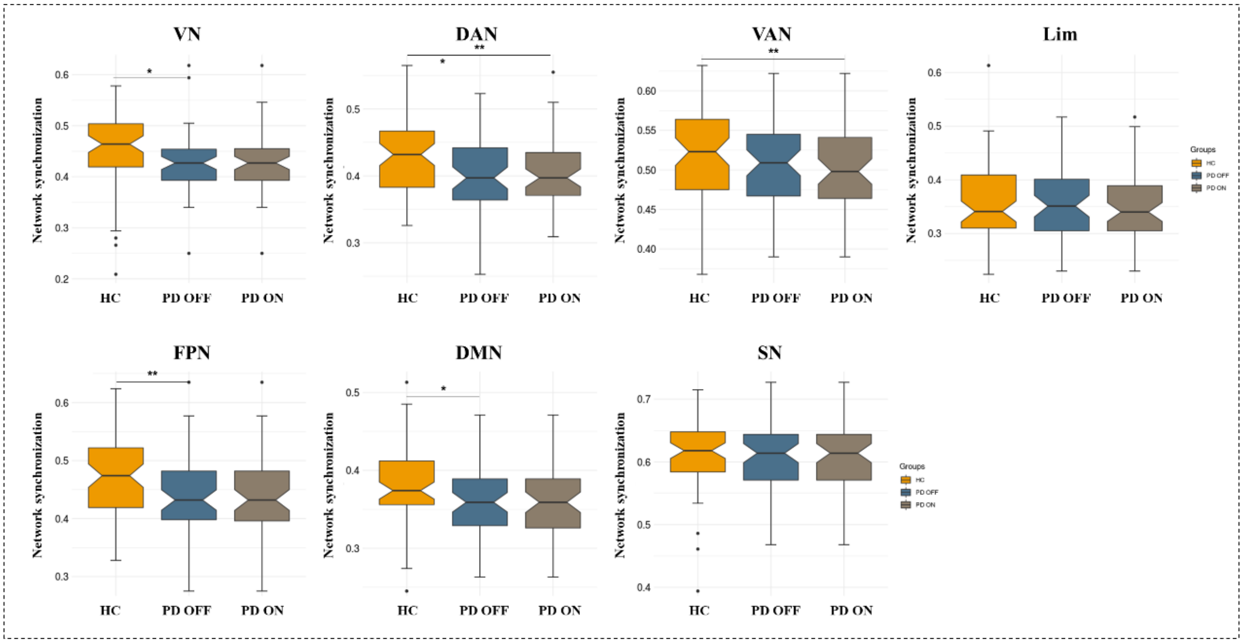

Supplement: Supplementary Material [file jpd-11-jpd212720-s001.pdf]
